# SecAODV: A Secure Healthcare Routing Scheme Based on Hybrid Cryptography in Wireless Body Sensor Networks

**DOI:** 10.3389/fmed.2022.829055

**Published:** 2022-07-21

**Authors:** Heon Jeong, Sang-Woong Lee, Mazhar Hussain Malik, Efat Yousefpoor, Mohammad Sadegh Yousefpoor, Omed Hassan Ahmed, Mehdi Hosseinzadeh, Amir Mosavi

**Affiliations:** ^1^Department of Fire Service Administration, Chodang University, Muan-gun, South Korea; ^2^Pattern Recognition and Machine Learning Lab, Gachon University, Seongnam, South Korea; ^3^HoD Computing and IT (CIT) Global College of Engineering and Technology, Muscat, Oman; ^4^Department of Computer Engineering, Dezful Branch, Islamic Azad University, Dezful, Iran; ^5^Department of Information Technology, University of Human Development, Sulaymaniyah, Iraq; ^6^Faculty of Civil Engineering, Technische Universität Dresden, Dresden, Germany; ^7^John von Neumann Faculty of Informatics, Óbuda University, Budapest, Hungary; ^8^Institute of Information Engineering, Automation and Mathematics, Slovak University of Technology in Bratislava, Bratislava, Slovakia

**Keywords:** wireless body sensor networks (WBSNs), Internet of things (IoT), secure routing, security, healthcare

## Abstract

In recent decades, the use of sensors has dramatically grown to monitor human body activities and maintain the health status. In this application, routing and secure data transmission are very important to prevent the unauthorized access by attackers to health data. In this article, we propose a secure routing scheme called SecAODV for heterogeneous wireless body sensor networks. SecAODV has three phases: bootstrapping, routing between cluster head nodes, and communication security. In the bootstrapping phase, the base station loads system parameters and encryption functions in the memory of sensor nodes. In the routing phase, each cluster head node calculates its degree based on several parameters, including, distance, residual energy, link quality, and the number of hops, to decide for rebroadcasting the route request (RREQ) message. In the communication security phase, a symmetric cryptography method is used to protect intra-cluster communications. Also, an asymmetric cryptography method is used to secure communication links between cluster head nodes. The proposed secure routing scheme is simulated in the network simulator version 2 (NS2) simulator. The simulation results are compared with the secure multi tier energy-efficient routing scheme (SMEER) and the centralized low-energy adaptive clustering hierarchy (LEACH-C). The results show that SecAODV improves end-to-end delay, throughput, energy consumption, packet delivery rate (PDR), and packet loss rate (PLR).

## 1. Introduction

**Figure 1:**
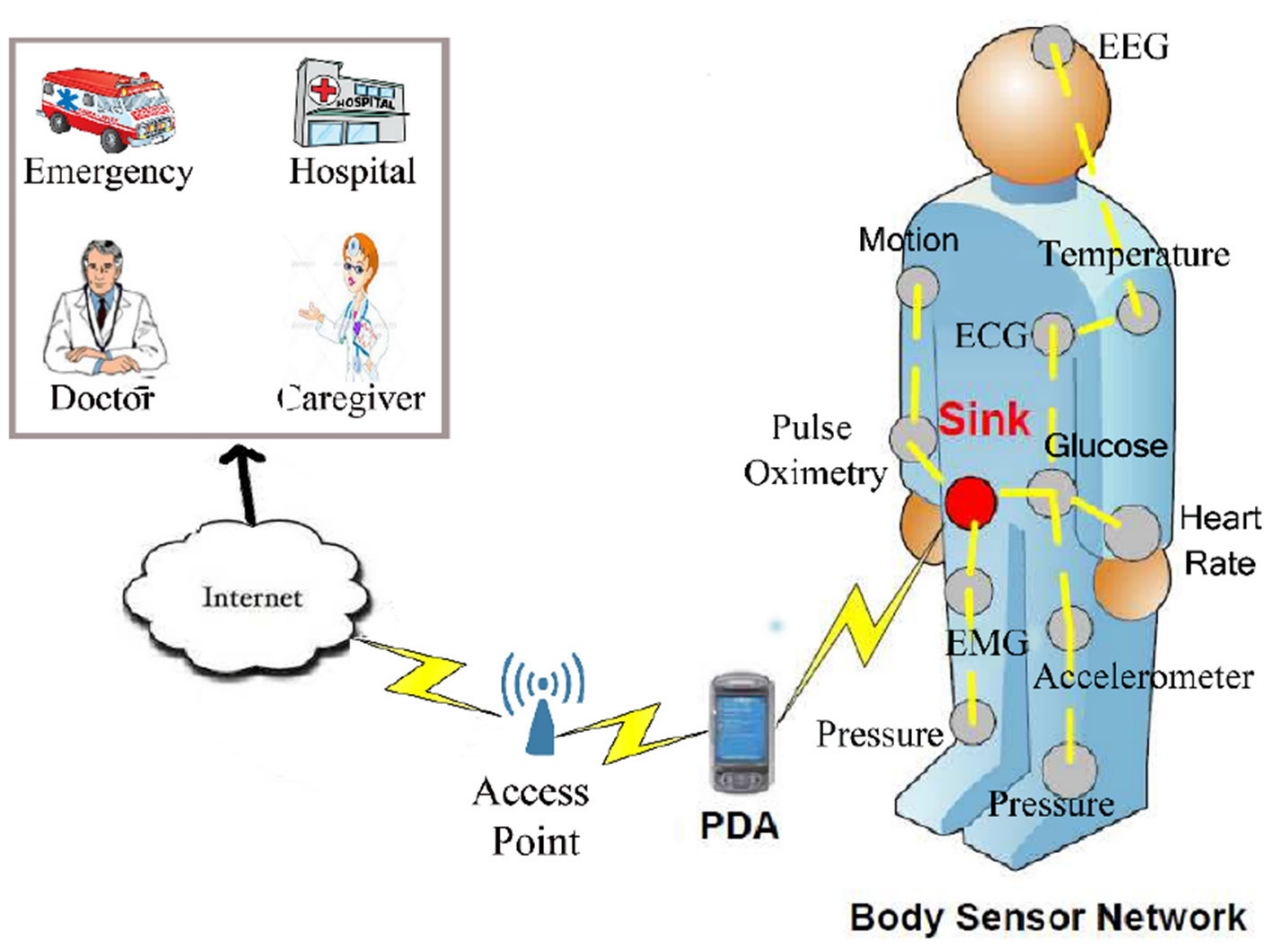
Wireless body network and its applications (9).

Recent advances in creating low-consumption electrical circuits for wireless communication allow us to produce small, low-consumption, and inexpensive equipment such as smart sensors. These intelligent sensors are devices that are installed on different objects to measure various parameters (1, 2). Sensors have different types, for example, thermal, magnetic, light, mechanical, and chemical. They are equipped with a small battery (3, 4). Recharging this battery is very difficult because sensors are usually scattered in insecure and inaccessible environments (5, 6). Therefore, these sensors have limited energy. When a number of sensor nodes monitor the human body and control human body activities to collect health data of individuals, they create a wireless body sensor network (WBSN) (7, 8), which is shown in Figure 1.

**Figure 2:**
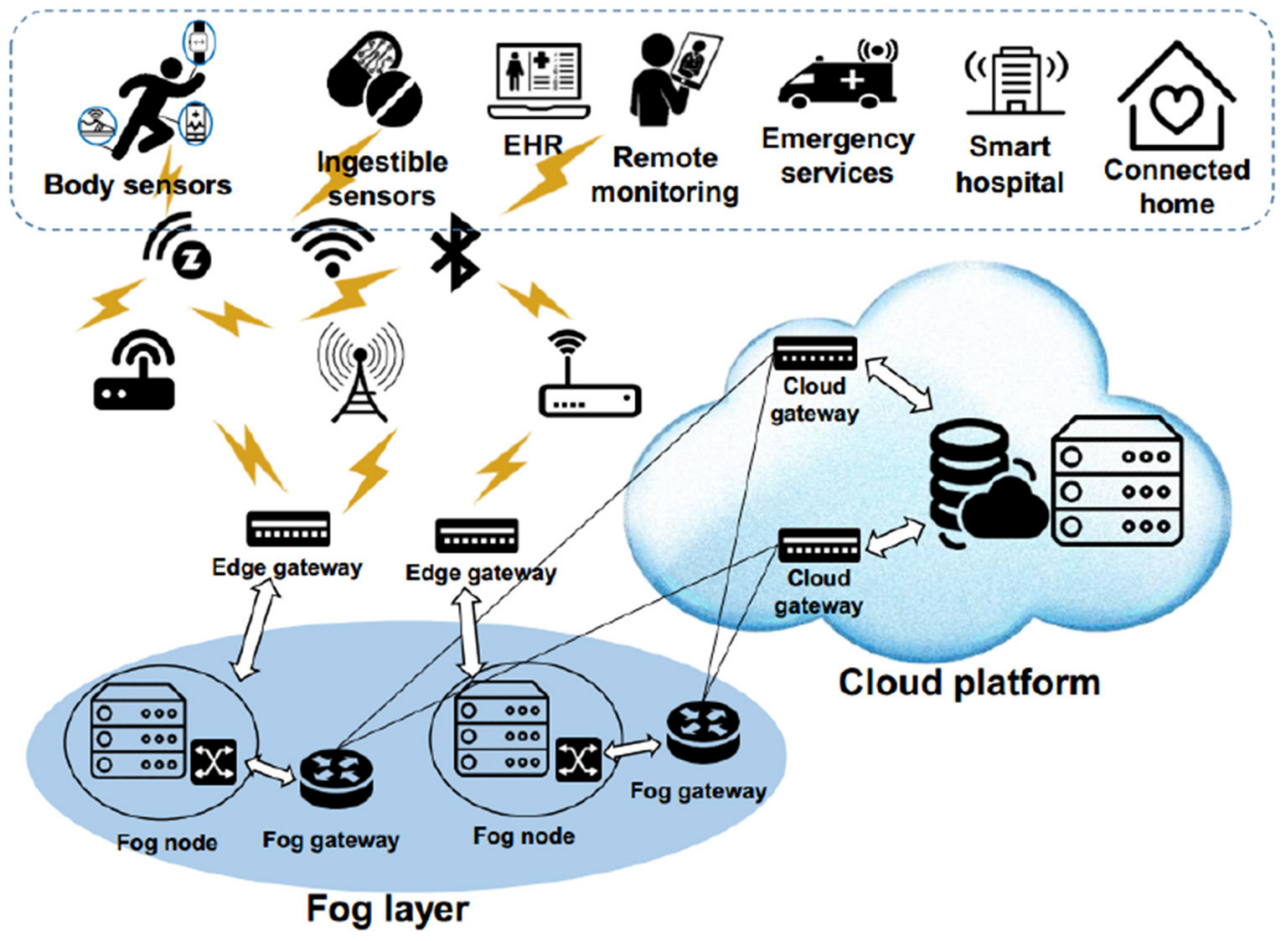
Various HIoT applications (24).

Today, this advanced and efficient technology has created promising opportunities for new technologies, such as the Internet of things (IoT) (10, 11). In the IoT, each object has a digital identifier and can communicate with other objects to provide services or receive the desired services (12, 13). In IoT, smart and small objects such as bulb switches, industrial machines (14), home equipment (15), meters, vehicles (16), and human body (17) are connected to each other using the IoT platform (18, 19). In this case, they present different services such as remote monitoring of the human body physiological data, monitoring physicians and patients in hospitals, medication management in hospitals, monitoring environmental conditions, monitoring irrigation, intelligent agriculture, smart homes, and controlling road traffic (20, 21). Healthcare Internet of Things (HIoT) is a new network, which combines IoT and WBSNs. HIoT monitors patients or the elderly in the family using sensor nodes to measure parameters such as blood sugar, heart rate, blood pressure, temperature, etc. To care and control respiratory patients, such as patients with COVID-19, sensor nodes can measure their vital signs and various parameters (22, 23). As a result, physicians and nurses can be aware of the patient's health status through processing this data. Also, crowded environments (such as streets, shopping malls, and so on) can be monitored by sensor nodes in terms of light, air pressure, magnetic field, sound, and vibration to assess their health. Figure 2 shows various HIoT applications.

In WBSNs, the routing process is very challenging because these networks have specific characteristics, such as resource constraints, unreliable communication, unsupervised operations, and lack of central management. In wireless sensor networks (WSNs), routing methods are categorized into five classes: data-centric routing, hierarchical routing, location-based routing, quality-of-service (QoS) aware routing, and secure routing. Moreover, communication security is known as a fundamental need in WBSNs because health data are very sensitive. If attackers change slightly the data, physicians analyze the wrong data and provide false treatment recommendations. On the other hand, patients tend to confidentially maintain their health information because if attackers achieve health data of patients, they may bring irreparable injuries to their personal and social life. A secure routing method should ensure data confidentiality, data integrity, authentication, and data availability when there are attacker nodes in the network. However, a secure routing scheme cannot guarantee all security requirements, but it must protect the network against routing attacks (25).

In this article, we propose a secure routing scheme called SecAODV for WBSNs. The purpose of this routing method is to improve energy consumption in the routing process and maintain security in communication links. In a secure routing scheme, the energy problem is very important because it affects directly the network lifetime. Therefore, when designing a secure routing method, it is necessary to reduce the energy consumption of sensor nodes. Security also has special importance in secure routing scheme in WBSNs because capturing sensor nodes by attackers affects negatively network performance. Cryptography is the most common method for maintaining data confidentiality. Symmetric key cryptography methods are desirable in terms of energy consumption, but they have a lower security level. In contrast, asymmetric key cryptography schemes guarantee better security level in the network, but they consume high energy. According to the items mentioned, SecAODV uses a hybrid key cryptography mechanism to utilize the benefits of both cryptography techniques and reduce their disadvantages. Our contributions are summarized as follows:

- We prioritize cluster head nodes (CHs) based on their degree to decide for sending the RREQ message. We calculate this degree based on the information available in the route request (RREQ) packet, including distance to the destination node, residual energy, link quality, and the number of hops. If their degree is greater than a threshold value, they rebroadcast the RREQ packet. Otherwise, the RREQ packet is deleted. This reduces communication overhead and network congestion, and balances energy consumption in the network.
- We use both key cryptography methods to secure communication links within the cluster and create a secure communication between cluster head nodes. CHs are responsible for producing the cluster key and sending it to cluster member nodes. The cluster key is a symmetric key, which is used to secure intra-cluster communications. Inter-cluster communications are also protected by asymmetric keys.
- We evaluate the performance of SecAODV and compare its results with SMEER and LEACH-C in terms of end-to-end delay, throughput, energy consumption, packet delivery rate (PDR), and packet loss rate (PLR).

In the following, the article is organized as follows: Section 2 presents the related works. In Section 3, the system model, including network model and attack model is explained. Section 4 describes the proposed secure routing scheme in detail. Section 5 analyses the security of SecAODV. Section 6 presents the simulation results of SecAODV. Finally, Section 7 concludes the article.

## 2. Related Works

Dhand et al. (26) presented the secure multi tier energy-efficient routing scheme (SMEER) for heterogeneous WSNs. The main goal of SMEER is to improve network security and reduce energy consumption in the network. In SMEER, sensor nodes are clustered using K-means algorithm and then the ant lion optimization algorithm (ALO) is used to select the best CH in each cluster. Clustering increases energy productivity, which boosts the network lifetime. Also, it improves the scalability of SMEER. In SMEER, an elliptic curve cryptographic (ECC) technique is used to secure data packets sent to the base station. Although, ECC increases energy consumption in the network, but it can guarantee better security. Also, the ALO algorithm causes high computational and communication overheads in the network.

Sun et al. (27) offered the secure routing protocol based on multi-objective ant-colony-optimization (SRPMA) in WSNs. The authors modify the ant colony algorithm to become a multi-objective routing algorithm. To achieve optimal solution, SRPMA considers two optimization objectives, including trust value and remaining energy of nodes to improve network lifetime and security. To evaluate trust of nodes, SRPMA introduces a trust evaluation model based on D-S evidence theory. Although, SRPMA only focuses on two parameters, including energy and trust in the routing process and ignores other parameters such as link quality and distance between nodes. This method does not use clustering technique and is not scalable. Moreover, ACO has high communication and computational overheads, which increase energy consumption and delay in the routing process.

Yang et al. (28) suggested a secure routing scheme based on blockchain and reinforcement learning (RLBC) in WSNs. RLBC includes two main parts: routing network and blockchain network. The blockchain network is responsible for making tamper-proof and trusted routing information because it traces this information. Also, the reinforcement learning-based routing algorithm selects paths through dynamic learning of nodes. In each hop, the path information is recorded in the blockchain. Therefore, if each hop includes a routing loop, the link is invalid, or the transmission rate is low, this algorithm reduces the probability of passing through this path. As a result, RLBC can dynamically discover efficient and reliable paths. However, the reinforcement learning algorithm and the blockchain network increase computational overhead and the time complexity of RLBC. Also, it increases delay in the network. Furthermore, RLBC does not consider the energy of nodes in the routing process. RLBC has led to unbalanced energy consumption in the network. Also, RLBC ignores the clustering process in the network and is not scalable.

Shi et al. (29) proposed a secure routing scheme called IASR for WSNs. This scheme uses an improved version of the Dijkstra algorithm to secure routes despite hostile nodes in the network. IASR selects next-hop nodes based on status and trust value. The trust value defines the attack probability based on node's behavior when sending the previous packet. Status combines remaining energy and distance to the sink node. Therefore, IASR produces an optimal route with minimum cost, which is secure against hostile attacks. Moreover, IASR does not require global information when selecting a secure route. This means that IASR acts based on local information. However, IASR does not consider the clustering process in the network and is not scalable. On the other hand, in IASR, there may be paths with a high delay that is not suitable.

Mehmood et al. (30) suggested a secure and low-energy zone-based routing scheme (SeLeZoR) for WSNs. SeLeZoR divides network nodes into several zones. Then, each zone includes a number of clusters with unequal sizes. Clusters far from the base station are larger than clusters close to BS. This improves scalability, balances energy consumption, and reduces traffic in the network. Cluster member nodes are responsible for sensing the environment and sending the collected data to its CH. This process is performed with the minimum transmission power determined by the received signal strength index (RSSI). The cluster head node (CH) encrypts these data packets using a secret key and sends them to its ZH. Then, ZH sends data to the BS using a secure and efficient mechanism. SeLeZoR introduces a key management system to guarantee secure communication between the network nodes. This system generates and distributes different keys for nodes in the network. Also, this scheme efficiently utilizes the transmission channel because it applies the time division multiple access (TDMA). However, SeLeZoR uses only the symmetric key cryptography technique. Furthermore, it does not describe the key management system in detail.

Perkins et al. (31) presented the *ad hoc* on-demand distance vector (AODV) for *ad hoc* networks. In AODV, each node maintains a routing table that saves the address of the next-hop node to reach the destination. When the source node wants to send its packet to the destination node and there is no valid route, it initiates the route discovery process to find a path. To ensure that the discovered paths are free-loop, and control packets include the newest information, AODV uses a sequence number. Therefore, nodes can detect duplicated control packets caused by the flooding process. Furthermore, AODV uses a route maintenance mechanism. In AODV, when the network is large, nodes may be delayed when discovering the path. In addition, link breakage in the route discovery process leads to a lot of delay and high bandwidth consumption.

Heinzelman et al. (32) introduced an improved version of the low-energy adaptive clustering hierarchy (LEACH) called centralized LEACH (LEACH-C). It uses a centralized clustering algorithm. In LEACH-C, each node sends information about its location and energy to the base station. BS is responsible for determining appropriate clusters and balancing energy consumption in the network. In order to achieve these goals, BS calculates the average energy of nodes. If a node has less energy than the average energy, it cannot be selected as the CH node in the current iteration. BS applies the simulated annealing algorithm to select CHs. This algorithm attempts to reduce energy consumed by non-CH nodes when sending data to CH. For this purpose, it minimizes the sum of the squared distances between non-CH nodes and the nearest CH. However, WSNs are more consistent with distributed algorithms because a centralized algorithm deals with the single point of failure issue and can be subjected to various attacks.

Sathya and Umadevi (33) offered a dynamic rate aware classified key distributional secure routing (DRCKDS) for WSNs. DRCKDS categorizes data based on its sensitivity and sensor nodes based on its importance. Then, this information is used to classify and distribute secret keys. This can reduce energy consumption in the network because data with low sensitivity requires less security. Each node maintains a neighboring table that includes information about neighboring nodes such as node type, number of transfers, number of re-transfer, and its location. This information is used when discovering paths between the source node and the destination node. However, DRCKDS did not explain the routing process exactly. Finally, DRCKDS calculates the secure route measure (SRM) for each path to select a secure route. This scale is based on the number of transfers and the number of re-transfers, which is performed by nodes in that path. However, DRCKDS does not explain how to calculate the trust value of nodes based on their behavior. Then, DRCKDS generates symmetric keys to secure the data transmission process and distributes these keys between the nodes in the path.

Mathapati et al. (34) proposed a secure routing method with multi-dimensional trust assessment for WSNs. This method presents a multi-dimensional trust assessment mechanism to evaluate the trust of nodes based on three dimensions include remaining energy, transmission pattern, and messages. Also, the authors introduce a trust recommender system to determine the compromised nodes and update the trust value. After determining the trust level of paths, a lightweight encryption system is used to provide security in the data transmission process. This reduces delay in this process. However, this method assumes that the network is not clustered and does not pay attention to scalability. In addition, this scheme does not consider parameters such as distance between the nodes, link quality, and energy in the routing process.

## 3. System Model

In this section, we describes the assumptions related to the network model and the attack model in SecAODV.

### 3.1. Network Model

**Figure 3:**
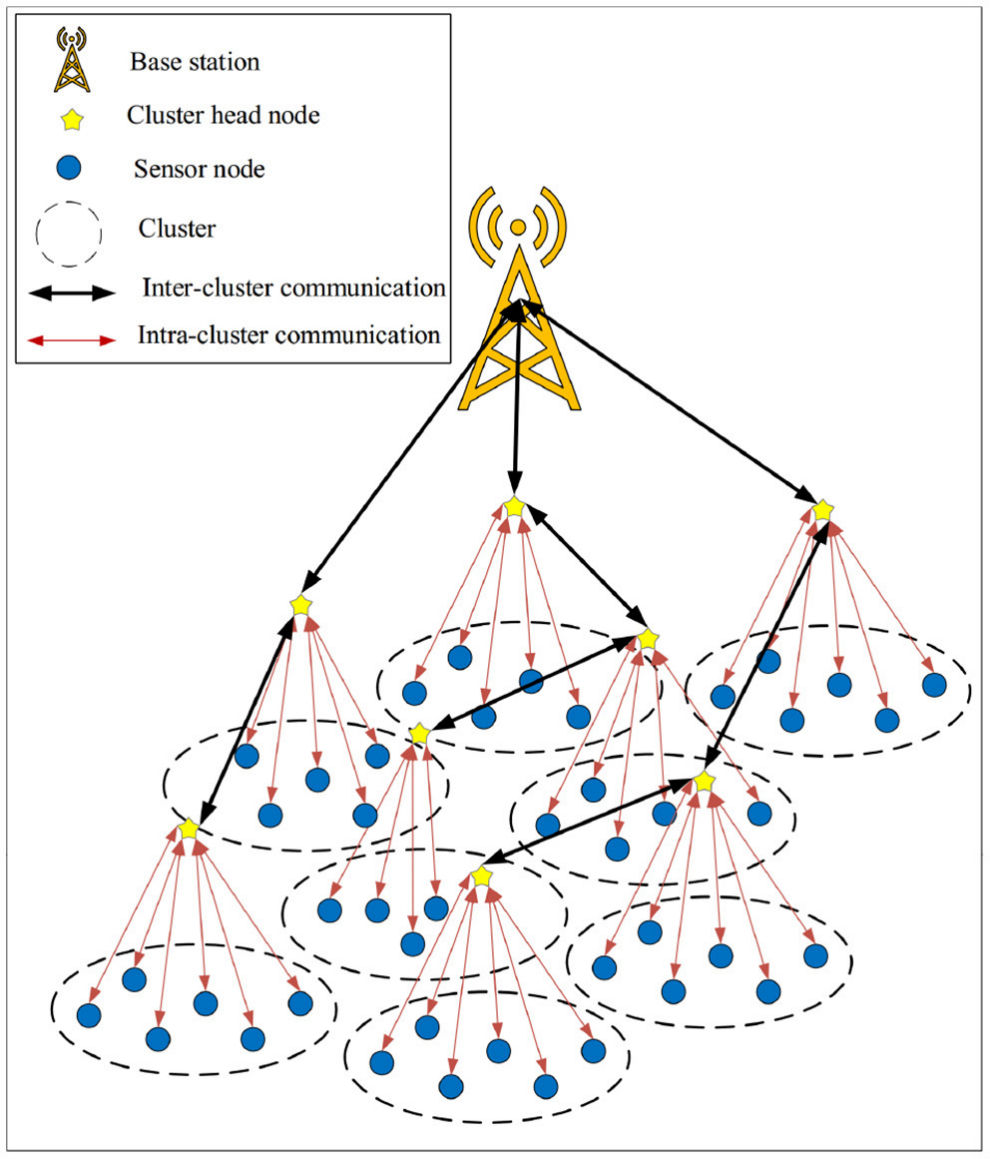
Network model in SecAODV.

In SecAODV, the network model is a heterogeneous wireless body sensor network. In this method, we assume that the network is clustered by the LEACH algorithm (35). In general, the network includes a powerful, reliable, and stable base station (BS), which has sufficient energy. Furthermore, it consists of a number of cluster head nodes (CHs) with more memory capacity and processing power than other sensor nodes, and a large number of normal sensor nodes with limited energy, memory capacity, and processing power, which act as cluster member nodes (CMs). CMs sense the environment and send the collected data to its CH node. Additionally, CHs receive data from their CMs and send them to the base station. Moreover, BS is responsible for processing data and managing the network. All network nodes know the location of BS in the network. Sensor nodes in the network (CHs or CMs) are static and equipped with a global positioning system (GPS). The network model is presented in Figure 3.

### 3.2. Attack Model

An adversary node launches various attacks in the network to disrupt its performance. In this article, we focus on eavesdropping and traffic analysis attacks. Adversary node listens to all communication links and accesses the information exchanged on those links. We assume that the adversary node can capture a sensor node in the network and achieve its confidential secret keys, ID, and other critical data. Then, the attacker misuses this confidential information to capture other nodes in the network and disrupt the normal network performance. Therefore, we focus on data confidentiality in this article.

## 4. Proposed Method

In SecAODV, the routing process has three phases, which are described in the following:

- Bootstrapping phase
- Routing phase
- Communication security phase

### 4.1. Bootstrapping Phase

In this phase, BS is assigned a unique identifier (ID) and a unique key (*k*_*i, BS*_) to each sensor node and loads a global key (*k*_*initial*_) in the memory of nodes. *k*_*initial*_ is used to protect communication between sensor nodes when bootstrapping the network. BS updates *k*_*initial*_ periodically or when sensor nodes die or are compromised by attackers. Then, BS encrypts this updated key using *k*_*i, BS*_ and unicasts the encrypted key only for valid nodes in the network. Moreover, BS loads some encryption parameters in the memory of CHs. Cluster head nodes use these parameters to secure communication channels in intra-cluster communication and inter-cluster communication. These parameters include:

- A key source for producing cluster key
- Public-private keys

Note that intra-cluster communication is secured by a symmetric key cryptography algorithm called the Rivest cipher 4 (RC4) and inter-cluster communication is protected using an asymmetric key cryptography algorithm called the elliptic curve cryptographic (ECC) technique. The RC4 cipher became the most widely used stream cipher due to its speed, simplicity, and efficient implementations in both software and hardware. It is a stream cipher with a secret key whose length is 1 to 256 bytes. Also, we select ECC because this method provides better security than traditional cryptography systems at a certain key size. As a result, ECC improves system security and can increase network performance by reducing key size and energy consumption to achieve an appropriate security level.

### 4.2. Routing Phase

When a cluster head node (for example, *CH*_*i*_) wants to send a data packet to another cluster head node (*CH*_*destination*_) and there is no path to *CH*_*destination*_, then *CH*_*i*_ begins the route discovery process. Algorithm 1 presents the pseudocode of the routing phase in SecAODV. In the following, we describe the various steps of this algorithm in details.

**Algorithm 1:** Route discovery process.

**Input:** *N*_*CH*_: Number of CHs in the network.
*CH*_*k*_: Cluster head nodes (*k* = 1, …, *N*_*CH*_).
**Output:** *Route*_*i*_ between *CH*_*source*_ and *CH*_*destination*_
**Begin**
1: ***CH*_*source*_:** Broadcast *RREQ message* for neighboring CHs (*CH*_*k*_);
2: **while** *ID*_*C*_*H*__*k*__≠*ID*_*C*_*H*__*destination*__ **do**
3: ***CH*_*k*_:** Calculate *Degree*_*k*_ based on Equation (2);
4: **if** *Degree*_*k*_≥*Degree*_*prev*−*hop*_ **then**
5: ***CH*_*k*_:** Broadcast *RREQ message* for neighboring CHs;
6: **end if**
7: **if** $Degree_{k}\geq \frac{1}{2}Degree_{prev-hop}$ **and** Any CH doesn't broadcast *RREQ message* at *Time*_*stop*_ **then**
8: ***CH*_*k*_:** Broadcast *RREQ message* for neighboring CHs;
9: **end if**
10: **if** Neighboring CHs don't broadcast *RREQ message* at $\frac{3}{2}Time_{stop}$ **then**
11: ***CH*_*k*_:** Broadcast *RREQ message* for neighboring CHs;
12: **end if**
13: **end while**
14: ***CH*_*k*_:** Send back *RREP message* to *CH*_*source*_;
**End**

**Figure 4:**
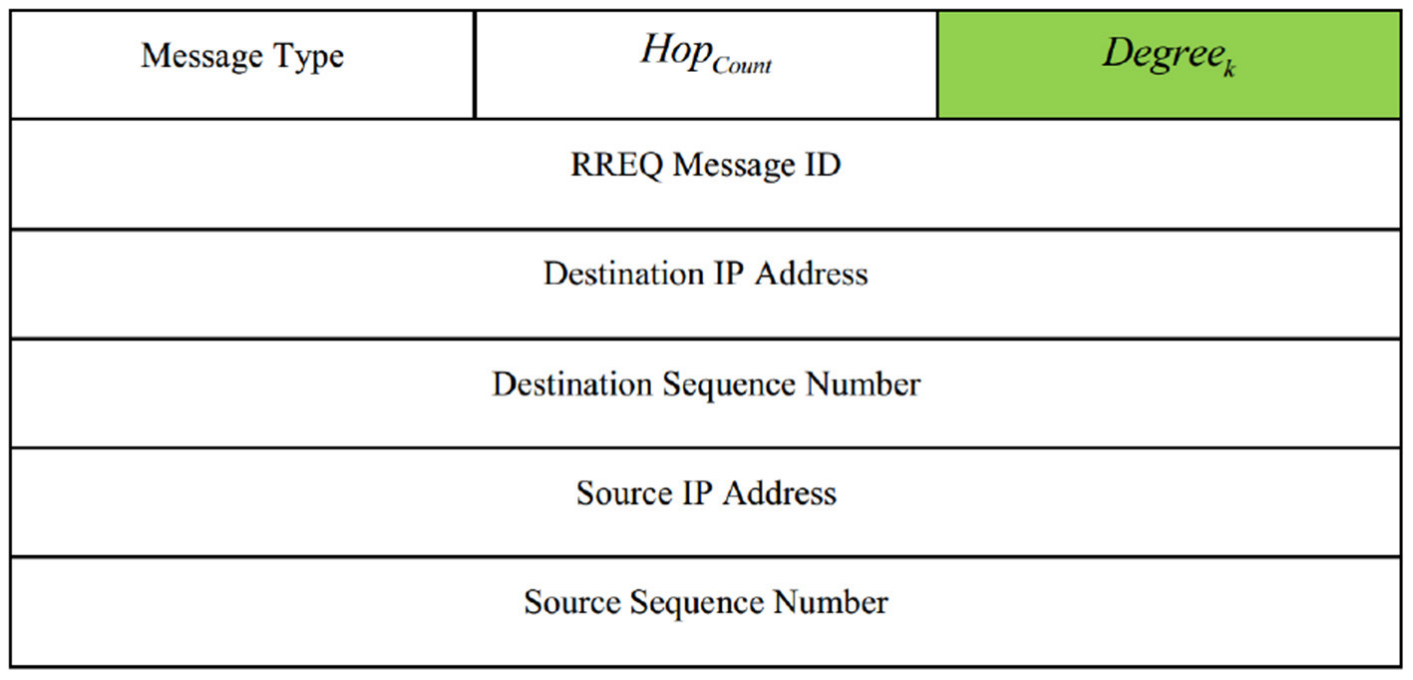
RREQ packet template.

In the first step (see line 1 of Algorithm 1), *CH*_*i*_ prepares a route request packet (RREQ) and adjusts its fields. Then, *CH*_*i*_ broadcasts this packet to its neighboring nodes. RREQ template is shown in Figure 4. As shown in this figure, the RREQ packet fields in SecAODV are similar to those in AODV. But there is a difference; the *Degree*_*k*_ field, which is shown in green color. This field indicates the degree of CH node that sends the RREQ packet.

After receiving the RREQ packet, neighboring cluster head nodes (for example, *CH*_*k*_) compute *Degree*_*k*_ based on four parameters, including distance to the destination node, residual energy, link quality, and the number of hops (see line 3 of Algorithm 1). *Degree*_*k*_ determines whether *CH*_*k*_ can replay the RREQ packet or not? *CH*_*k*_ can replay the RREQ packet only when it meets one of the following three modes:

- **Mode 1:** The degree of *CH*_*k*_ is greater than the degree of the previous-hop node (*CH*_*prev*−*hop*_), i.e., *Degree*_*k*_≥*Degree*_*pev*−*hop*_. Note that *Degree*_*pev*−*hop*_ is inserted into the RREQ packet (see lines 4–6 of Algorithm 1).
- **Mode 2:** The degree of *CH*_*k*_ is more than half degree of *CH*_*prev*−*hop*_, i.e., $Degree_{k}\geq \frac{1}{2}Degree_{pev-hop}$ and none of neighboring CHs rebroadcast the RREQ packet at the time *Time*_*stop*_ (see lines 7–9 of Algorithm 1).
- **Mode 3:** A period of time equal to $\frac{3}{2}Time_{stop}$ is finished and at this time, *CH*_*k*_ listens to the communication channel and understands that none of neighboring CHs rebroadcast the RREQ packet (see lines 10–12 of Algorithm 1).

Before rebroadcasting the RREQ packet, *CH*_*k*_ updates *Hop*_*count*_ field and adds one unit to it. Also, it updates the *Degree*_*k*_ field and inserts its degree in this field. This process continues until the RREQ packet reaches the destination node.

*Degree*_*k*_ is calculated according to four parameters:

- Distance between *CH*_*k*_ and *CH*_*destination*_ (*d*_*k, destination*_): The purpose of choosing this parameter is that if the distance between *CH*_*k*_ and *CH*_*destination*_ is low, then *CH*_*k*_ has more chance to be selected as the next-hop node (*CH*_*next*−*hop*_). In this regard, whenever *CH*_*k*_ receives the RREQ packet, it calculates its Euclidean distance to *CH*_*destination*_ based on Equation (1):
  (1) $$\begin{array}{r} d_{k,destination}=\sqrt{{\left(x_{k}-x_{des}\right)}^{2}+{\left(y_{k}-y_{des}\right)}^{2}} \end{array}$$
  Where, (*x*_*k*_, *y*_*k*_) and (*x*_*des*_, *y*_*des*_) indicate the spatial coordinates of *CH*_*k*_ and *CH*_*destination*_, respectively.
- Remaining energy (*e*_*k*_): The purpose of choosing this parameter is that if *CH*_*k*_ has a lot of energy, then this node has gained a higher score for participating in the route formation process and have more chance to be selected as *CH*_*next*−*hop*_. CHs are aware of their remaining energy (*e*_*k*_) at any moment.
- Link quality (*q*_*k, prev*−*hop*_): The purpose of this parameter is to create high-quality routes. Therefore, if the quality of the link between *CH*_*k*_ and *CH*_*prev*−*hop*_ is high, then this node gains more score to be selected as *CH*_*next*−*hop*_. The link quality is determined based on the received signal strength indication (RSSI) (36). Note that RSSI is a register installed on radio transmitters/receivers. RSSI calculates the signal strength when receiving the RREQ packet (37). Researchers show that more RSSI improves PDR. Furthermore, RSSI is stable at the short period of time (about 2 s) and its standard deviation is less than 1*dBm* (38). For this reason, this indicator can be used to estimate link quality. When receiving the RREQ packet, *CH*_*k*_ estimates the quality of the link between itself and *CH*_*prev*−*hop*_.
- Number of hops (*Hop*_*Count*_): This parameter indicates the number of hops from the source node to the current CH node. Note that in the route formation process, routes with lower hops are better. This parameter is inserted in the RREQ packet and is added one unit in each hop.

Finally, *Degree*_*k*_ is calculated according to Equation (2):


(2) $$\begin{array}{l} Degree_{k}=\left(\frac{q_{k,prev-hop}-q_{\min}}{q_{\max}-q_{\min}}\right)+\left(\frac{e_{k}-e_{\min}}{e_{\max}-e_{\min}}\right)\\ \;+\left(1-\frac{Hop_{Count}}{N-1}\right)+\left(1-\frac{d_{k,destination}}{d_{\max}}\right) \end{array}$$

Where, *q*_*k, prev*−*hop*_ is the quality of the link between *CH*_*k*_ and *CH*_*prev*−*hop*_. According to (36), when RSSI has more value, it increases PDR, which indicates a better link quality. If *q*_max_ = *RSSI* = 87*dBm*, then *PDR* = 99%. Also, when *q*_min_ = *RSSI* = 0*dBm*, then *PDR* = 0. Furthermore, *e*_*k*_ represents the residual energy of *CH*_*k*_, *e*_max_ indicates the initial energy of CH nodes and *e*_min_ = 0.1*e*_max_. *Hop*_*Count*_ is the number of hops from the source node to *CH*_*k*_. *N* indicates the number of sensor nodes in the network, *d*_*k, destination*_ is the Euclidean distance between *CH*_*k*_ and *CH*_*destination*_. *d*_max_ is determined based on the network size. Suppose that the network size is equal to *n*×*m*, then $d_{\max}=\sqrt{n^{2}+m^{2}}$.

After the RREQ packet reaches *CH*_*destination*_, this node prepares a route reply packet (RREP) and sends back it to *CH*_*source*_ according to the determined path (see line 14 of Algorithm 1). After receiving the RREP packet, the source node inserts information about this path in its routing table. *CH*_*source*_ uses the route to send its data to *CH*_*destination*_. Note that SecAODV uses a route maintenance process similar to AODV. The purpose of the route maintenance process is that a node ensures that the paths in its routing table are valid and when a route failure occurs, the node updates this failed path.

### 4.3. Communication Security Phase

In this phase, we describe how to secure communication links within the cluster and how to protect communication links between CH nodes. As mentioned in Section 4.1, we have used the RC4 cipher as a symmetric key cryptography method due to its speed, simplicity, and efficient implementations in both software and hardware.

#### 4.3.1. Secure Intra-Cluster Communication

To provide security in communication links between cluster member nodes, we use a symmetric key cryptography method. Algorithm 2 presents the pseudocode of the secure intra-cluster communication process. In the following, we describe the steps of this algorithm.

**Algorithm 2:** Secure intra-cluster communications.

**Input:** *N*_*CM*_: Number of CMs in the cluster *i*.
*CH*_*i*_: Cluster head node corresponding to the cluster *i*.
*CM*_*j*_: CM nodes in the cluster *i* so that (*j* = 1, …, *N*_*CM*_).
**Output:** *k*_*cluster*_: Cluster key
**Begin**
1: ***CH*_*i*_:** Generate *k*_*cluster*_;
2: ***CH*_*i*_:** Encrypt *k*_*cluster*_ using *k*_*initial*_;
3: ***CH*_*i*_:** Broadcast the encrypted *k*_*cluster*_ for all CMs;
4: ***CM*_*j*_:** Decrypt the message and extract *k*_*cluster*_;
5: **if** *CM*_*j*_ wants to securely sent its data to *CH*_*i*_ **then**
6: ***CM*_*j*_:** Encrypt its data packet (*Data*_*C*_*M*__*j*__) using *k*_*cluster*_;
7: ***CM*_*j*_:** Send the encrypted data packet to *CH*_*i*_;
8: ***CH*_*i*_:** Decrypt the packet using *k*_*cluster*_ and extract *Data*_*C*_*M*__*j*__;
9: **end if**
**End**

The CH node (for example, *CH*_*i*_) is responsible for generating the cluster key and sending it to the cluster member nodes (for example, *CM*_*j*_). After creating clusters and determining their members, *CH*_*i*_ randomly selects a key from its key source, which is loaded in the memory of CHs before distributing nodes in the network (see line 1 of Algorithm 2).

Then, *CH*_*i*_ encrypts this cluster key (*k*_*cluster*_) using *k*_*initial*_ and broadcasts the encrypted key to *CM*_*j*_ (see lines 2-3 of Algorithm 2). This process is expressed in Equation (3).


(3) $$\begin{array}{r} CH_{i}\to *:Encrypt_{k_{initial}}\left(k_{cluster},ID_{CH_{i}}\right) \end{array}$$

When *CM*_*j*_ receives this message, it decodes this message using *k*_*initial*_, confirms the ID of *CH*_*i*_ and extracts *k*_*cluster*_ (see line 4 of Algorithm 2). This process is performed according to Equation (4).


(4) $$\begin{array}{r} CM:Decrypt_{k_{initial}}\left(k_{cluster},ID_{CH_{i}}\right) \end{array}$$

Therefore, *CM*_*j*_ encrypts its messages (*Data*_*C*_*M*__*j*__) using *k*_*cluster*_ and sends the encrypted message to *CH*_*i*_ (see lines 5-7 of Algorithm 2). The message encryption process is presented in Equation (5):


(5) $$\begin{array}{r} CM_{j}\to CH_{i}:Encrypt_{k_{cluster}}\left(Data_{CM_{j}},ID_{CM_{j}}\right) \end{array}$$

When *CH*_*i*_ receives the encrypted message from *CM*_*j*_, it performs the decryption process, confirms the ID of *CM*_*j*_ and extracts *Data*_*C*_*M*__*j*__ from the data packet (see line 8 of Algorithm 2). This process is shown in Equation (6):


(6) $$\begin{array}{r} CH_{i}:Decrypt_{k_{cluster}}\left(Data_{CM_{j}},ID_{CM_{j}}\right) \end{array}$$

**Figure 5:**
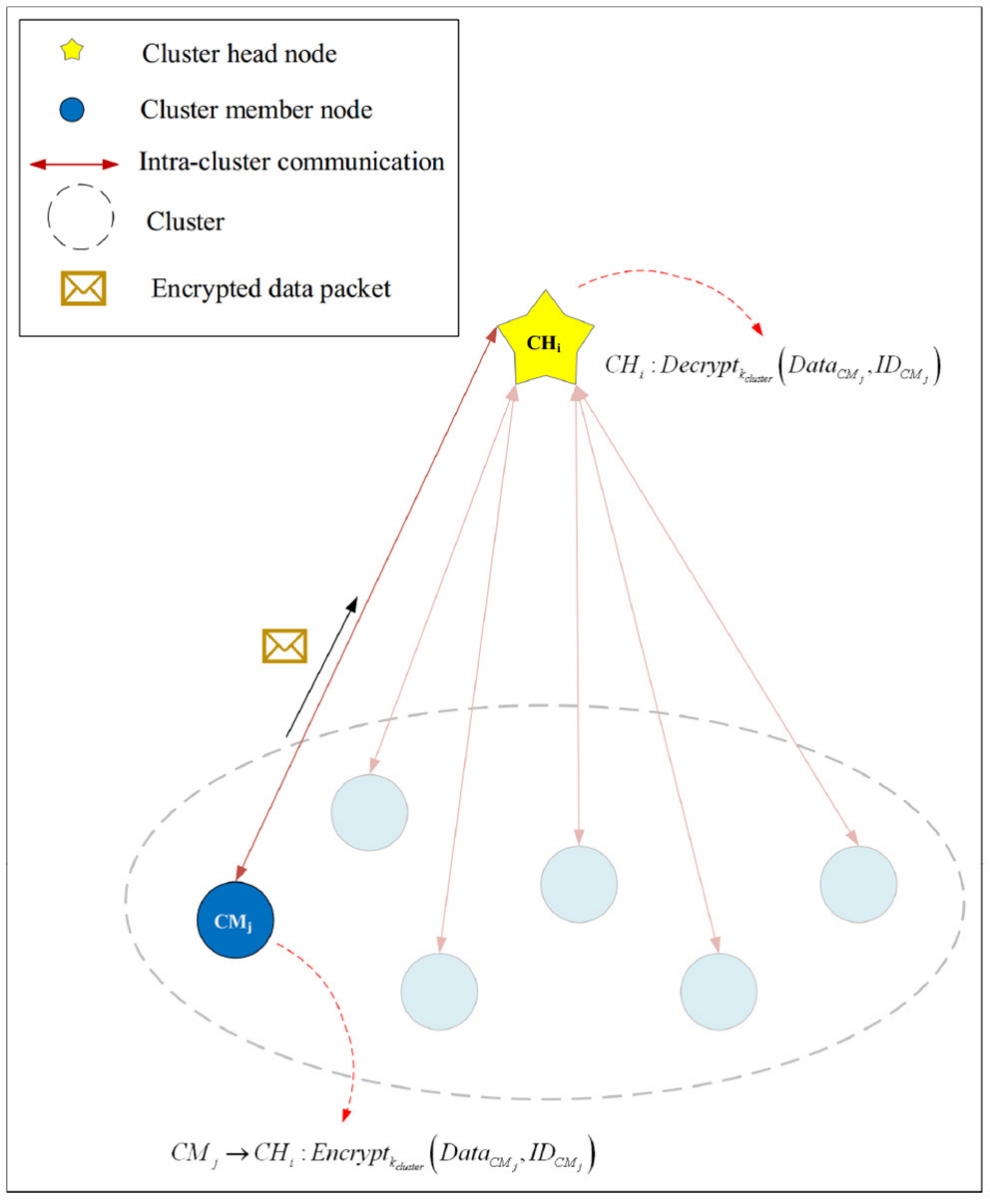
Secure intra-cluster communication.

This process is shown in Figure 5.

#### 4.3.2. Secure Inter-cluster Communication

CHs use an asymmetric key cryptography method called ECC to secure their communications. As stated in the bootstrapping phase, the base station produces the public-private keys (*k*_*pub*_−*k*_*pri*_) and loads them in memory of CHs before launching the network. CHs use this key to encrypt their messages. Algorithm 3 presents the pseudocode of the secure inter-cluster communication process. In the following, we describe the steps of this algorithm.

**Algorithm 3:** Secure inter-cluster communications.

**Input:** *CH*_*i*_: Cluster head node corresponding to the cluster *i*.
*CH*_*j*_: Cluster head node corresponding to the cluster *i*.
**Output:** Creating a secure inter cluster communication between *CH*_*i*_ and *CH*_*j*_.
**Begin**
1: ***CH*_*i*_:** Broadcast its public key (*k*_*pu*_*b*__*i*__) for all CHs;
2: **if** *CH*_*i*_ wants to securely send its data to *CH*_*j*_ **then**
3: ***CH*_*i*_:** Encrypt *Data*_*C*_*H*__*i*__ using *k*_*pu*_*b*__*j*__;
4: ***CH*_*i*_:** Send the encrypted data packet to *CH*_*j*_;
5: ***CH*_*j*_:** Decrypt the message using *k*_*pr*_*i*__*j*__ and extract *Data*_*C*_*H*__*i*__;
6: **end if**
**End**

After launching the network, each CH node shares its public key with its neighboring CHs and maintains its private key (see line 1 of Algorithm 3).

When *CH*_*i*_ wants to securely send *Data*_*C*_*H*__*i*__ to *CH*_*i*_, it encodes this message using the public key of *CH*_*i*_ (*k*_*pu*_*b*__*j*__) (see lines 2–4 of Algorithm 3). This process is presented in Equation (7):


(7) $$\begin{array}{r} CH_{i}\to CH_{j}:Encrypt_{k_{pub_{j}}}\left(Data_{CH_{i}},ID_{CH_{i}}\right) \end{array}$$

When *CH*_*j*_ receives this encrypted message from *CH*_*i*_, it decrypts this message using its private key (*k*_*pr*_*i*__*j*__) and extracts its information (see line 5 of Algorithm 3). This process is expressed in Equation (8).


(8) $$\begin{array}{r} CH_{j}:Decrypt_{k_{pri_{j}}}\left(Data_{CH_{i}},ID_{CH_{i}}\right) \end{array}$$

**Figure 6:**
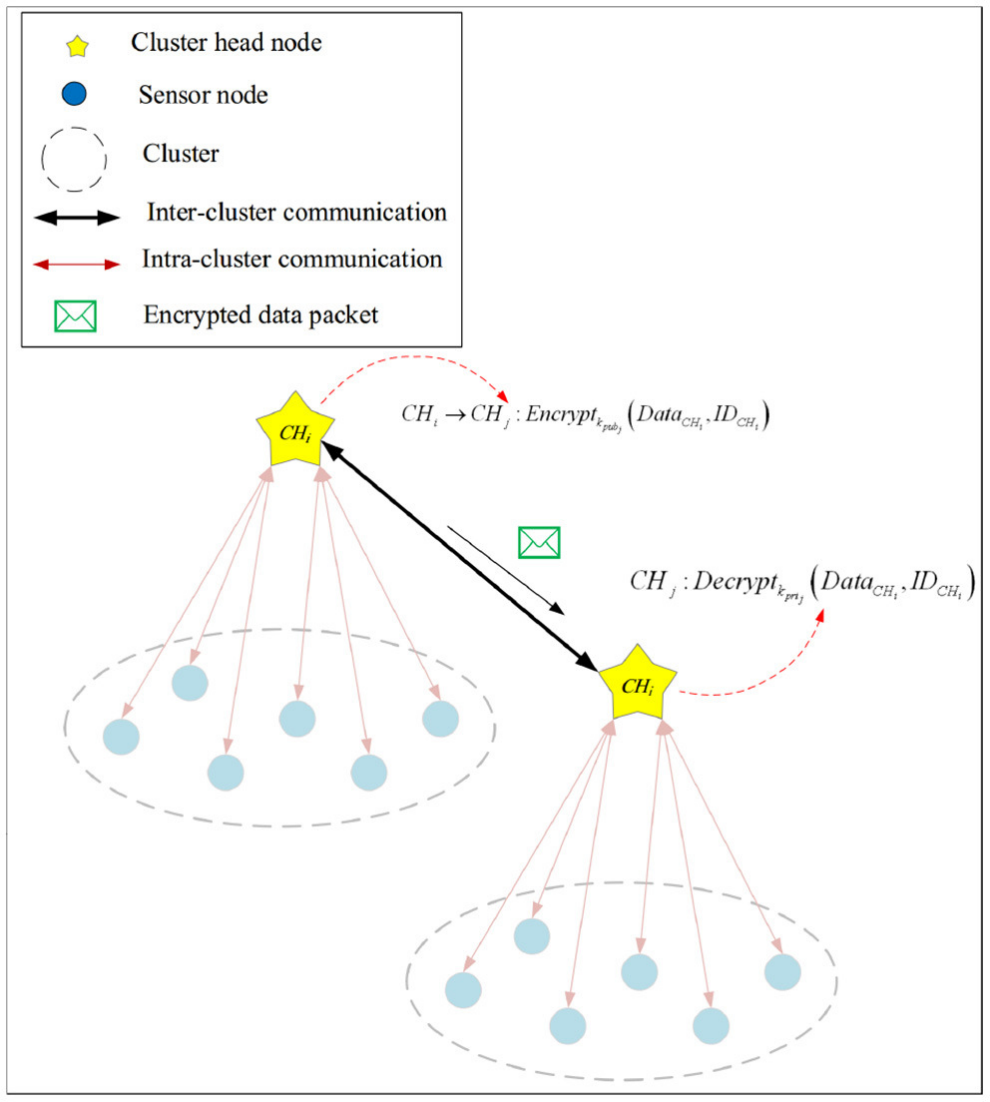
Secure inter-cluster communication.

Also, Figure 6 displays this process.

## 5. Security Analysis

In this section, we discuss the security of SecAODV briefly. Note that SecAODV uses a symmetric encryption technique to create a secure connection between cluster member nodes in a cluster. Also, CHs use the ECC encryption technique to protect their communications. Data confidentiality ensures that attackers cannot access sensitive information. SecAODV guarantees data confidentiality because data is encrypted in the data transmission process in a cluster. Therefore, an attacker cannot access the content of the encrypted data without knowing the cluster key. Attackers do not have access to this key because CHs produce this key and securely send the cluster key to CMs. The cluster key distribution is secured using *k*_*initial*_. On the other hand, attacker cannot decrypt data exchanged between CHs because they do not know the private key of CHs. Obtaining all private keys is not simple because each CH is aware of its private key and does not know the private key of other CHs. These keys are only available to the BS. Therefore, an attacker must compromise all CH nodes in the network to access their data and this is impossible. Therefore, our proposed method guarantees data confidentiality. This proves that SecAODV has a successful performance against attacks, such as eavesdropping and analytic analysis, because according to the mentioned items, if a hostile node listens to communication channels between two nodes, it cannot access information exchanged in the network. On the other hand, the proposed method has a successful performance against the capture node attack because SecAODV is a clustering-based routing method. Therefore, if an attacker compromises a cluster member node, this attack affect locally inside the cluster, and other communications are secure in the whole network. In addition, if the attacker captures a CH node, it achieves only its information and cannot disrupt secure communications between other CHs.

## 6. Simulation of the Proposed Method

In this section, we evaluate the performance of SecAODV. First, the proposed routing scheme is simulated using the NS-Allinone-2.35 simulator. Then, the simulation results are compared with two routing methods, including SMEER (26) and LEACH-C (32). In the simulation process, we assume that there are 100 sensor nodes, which are randomly scattered in the network with a size of 2, 500 × 50*m*^2^. These nodes do not move. The BS is located at the network center. The packet size is 1024 bits. The initial energy of normal sensor nodes and CH nodes are considered 0.5 and 1*J*, respectively. Furthermore, we consider the simulation time equal to 30 s. Other simulation parameters are summarized in Table 1. In the simulation process, we compare our scheme with SMEER and LEACH-C in terms of end-to-end delay, throughput, energy consumption, packet delivery rate (PDR), and packet loss rate (PLR).

**Table 1:** Simulation parameters.

**Parameter**	**Value**
Simulator	NS-2.35
Network size	50 × 2, 500*m*^2^
Location of BS	Network center
Total number of sensor nodes	100
Initial energy of CHs	1 J
Initial energy of sensor nodes	0.5 J
Antenna	Omni-Antenna
Packet size	1024 bit
Mac protocol	IEEE 802.11
Simulation time	30 s
Black hole nodes	5

### 6.1. End-To-End Delay

This parameter is defined as the sum of the time required to deliver the data packet to the recipient node. This parameter is obtained using Equation (9).


(9) $$\begin{array}{l} End-to-end\;delay\\ \;=\frac{Sum\;of\;time\;taken\;to\;deliver\;packet\;in\;receiver}{Noumber\;of\;packet\;received\;by\;receiver} \end{array}$$

**Figure 7:**
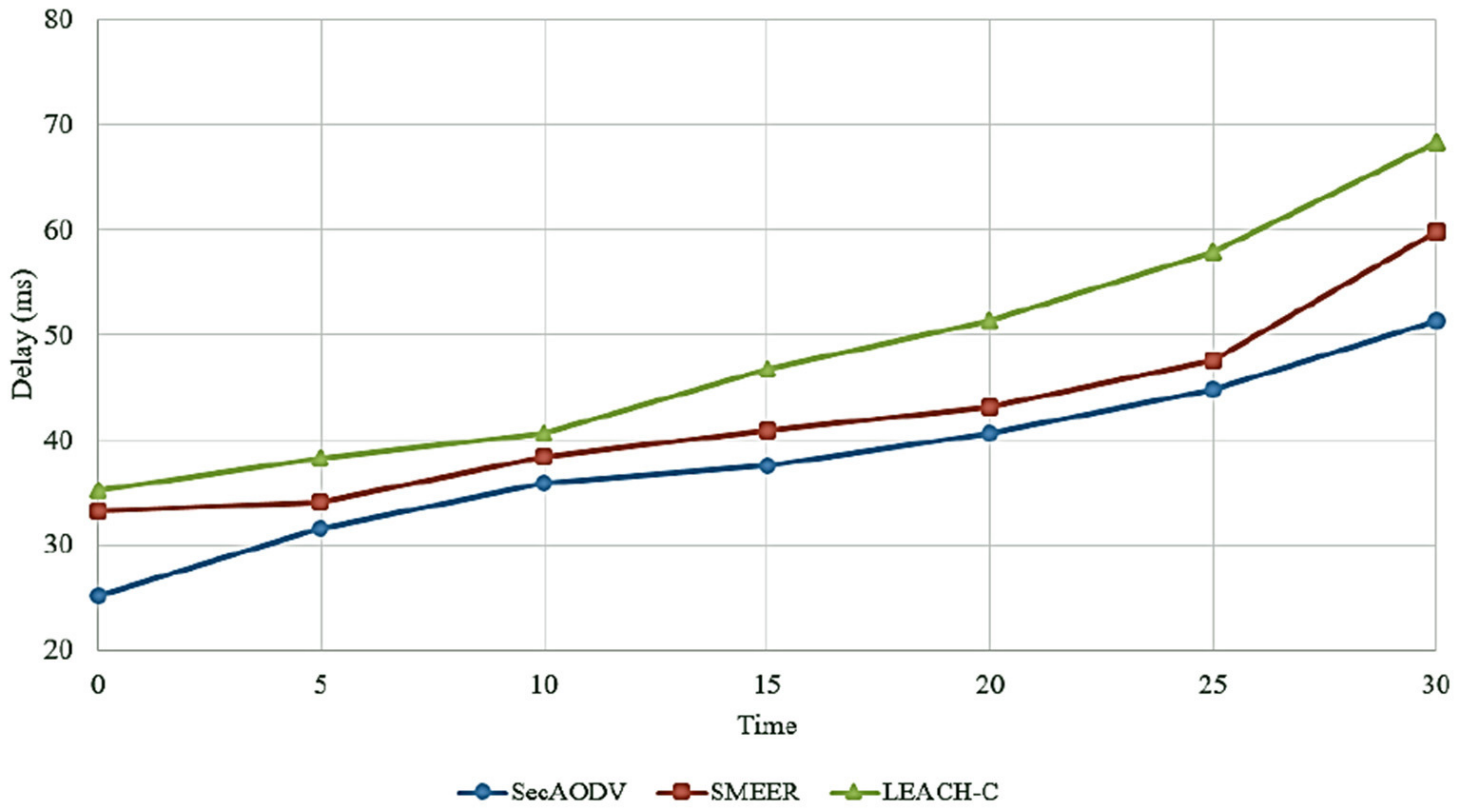
Comparison of different method in terms of delay.

Figure 7 compares end-to-end delay in different routing methods. As shown in this figure, SecAODV has the lowest delay in comparison with other routing methods. On average, our scheme decreases delay by 10.07 and 21.04% compared to SMEER and LEACH-C, respectively. As a result, SecAODV performs the data transmission process more quickly. This issue has several reasons: (1) SMEER uses only asymmetric encryption method to secure the data transfer process. This increases delay in this process. While SecAODV uses a hybrid cryptography scheme. In our proposed method, the symmetric key cryptography is used to build secure communication between cluster member nodes. So that CMs encrypt their data using the cluster key and send it to its CH. Also, the secure connection between CHs is guaranteed using an asymmetric key cryptography technique. (2) SMEER uses the ALO algorithm in the clustering process. This increases highly computational overhead and needs high iterations to find optimal response. These items increase delay and weaken the network performance. Additionally, LEACH-C uses the simulated annealing algorithm in the clustering process. It increases computational overhead and delay. In contrast, SecAODV utilizes the LEACH algorithm for clustering. It is faster than the other two methods. (3) SecAODV considers link quality and energy of the nodes. Therefore, it can create more stable routes than SMEER and LEACH-C. This reduces route failure. As a result, our scheme lowers delay in the routing process.

### 6.2. Throughput

This parameter is defined as the ratio of data packets received at the receiver node to delay required for transferring these packet. This parameter is calculated based on Equation (10):


(10) $$\begin{array}{r} Throughput=\frac{Number\;of\;packets\;received}{Delay} \end{array}$$

**Figure 8:**
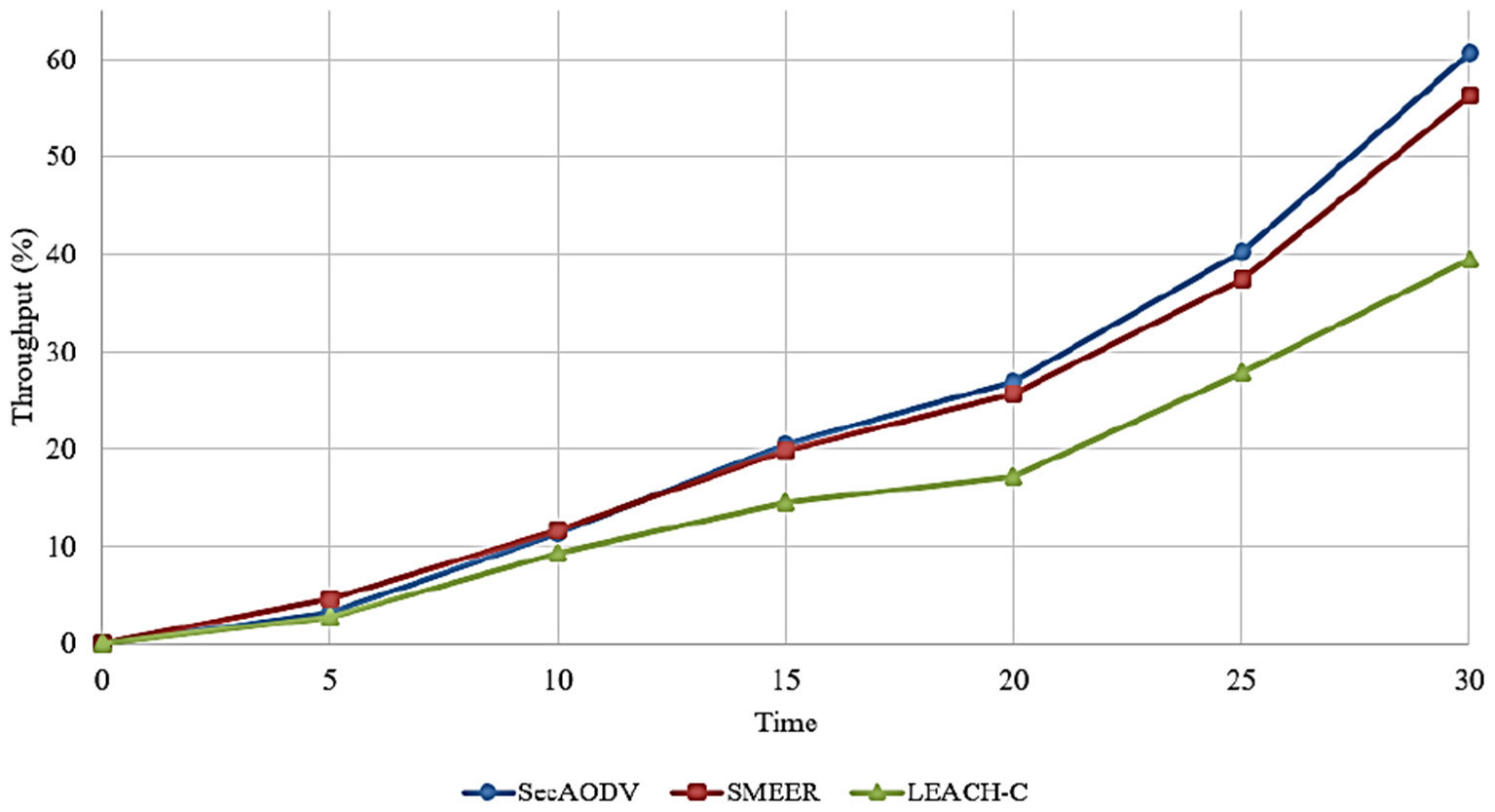
Comparison of different methods in terms of throughput.

Figure 8 compares different routing methods in terms of throughput. As shown in this figure, SecAODV has the best throughput compared to other routing methods because it improves throughput by 4.83 and 46.85% compared to SMEER and LEACH-C, respectively. This is because SecAODV has less delay than other routing methods in the data transmission process. We presented its reasons in Section 6.1. Secondly, in the routing process, SecAODV attempts to find high-energy nodes for creating paths. Furthermore, it creates high-quality routes with fewer hops. As a result, SecAODV facilitates the data transfer process and improves throughput.

### 6.3. Energy Consumption

**Figure 9:**
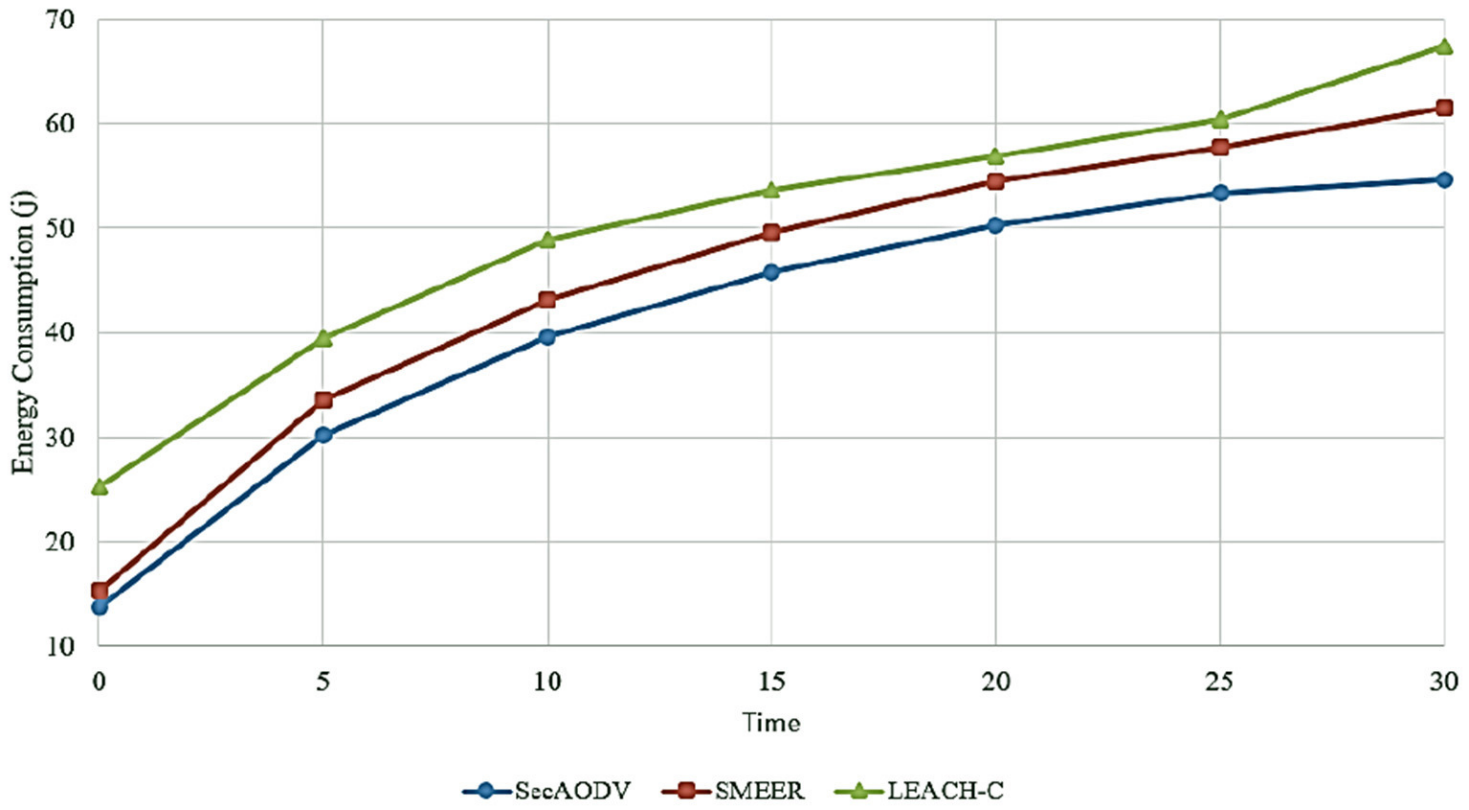
Comparison of different methods in terms of energy consumption.

This parameter is expressed as the sum of the required energy for receiving a packet and the required energy for sending the packet in the data transfer process. Figure 9 compares different routing methods in terms of energy consumption. As shown in this figure, SecAODV has the lowest energy consumption compared to other methods because it has reduced energy consumption by 8.82 and 18.31% compared to SMEER and LEACH-C, respectively. This has several reasons: firstly, in LEACH-C, CHs communicate with the BS in a single-hop manner. This increases energy consumption dramatically. On the other hand, SMEER performs the routing process in a multi-hop manner. This improves energy consumption. However, SMEER considers two parameters, including distance and the angle between nodes when selecting the next-hop node. Choosing more appropriate parameters can improve the performance of this routing method. In SecAODV, CHs communicate with the BS in a multi-hop manner. Also, it considers various parameters, including energy, distance, link quality, and the number of hops, when selecting the next-hop node. As a result, it creates more stable paths and reduces the packet loss rate. This improves energy consumption in the data transmission process.

### 6.4. Packet Loss Rate

This parameter is defined as the percentage of data packets that are not reached at the destination. This parameter is obtained according to Equation (11).


(11) $$\begin{array}{r} PLR=\frac{\overset{n}{\underset{i=1}{\sum }}P_{l}}{\overset{n}{\underset{i=1}{\sum }}P_{s}}\times 100 \end{array}$$

Where, *P*_*l*_ is packets that are not reached at the destination and *P*_*s*_ is packets sent by the source node.

**Figure 10:**
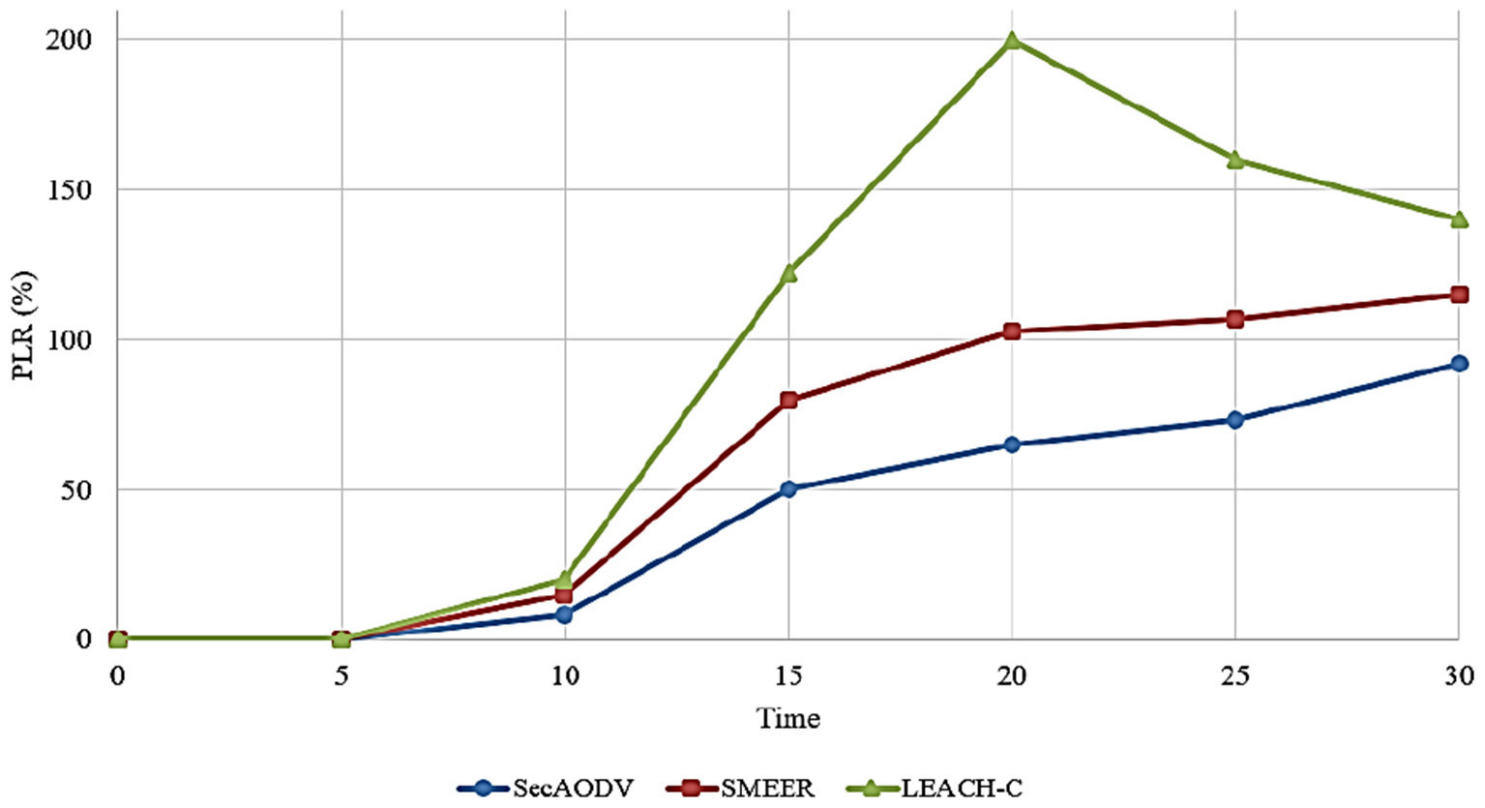
Comparison of different methods in terms of PLR.

Figure 10 compares different routing methods in terms of PLR. As shown in this figure, SecAODV has the lowest PLR compared to others because it has reduced PLR by 31.43 and 55.14% compared to SMEER and LEACH-C, respectively. LEACH-C has the worst PLR because CHs has high communication overhead and consume a lot of energy. They must receive data from its CM nodes and send directly the data to the BS. This can increase the packet loss rate. On the other hand, SMEER considers only two parameters, including distance and angle between neighbor nodes in the route discovery process. While, considering energy and link quality is very important. Therefore, SMEER may create unstable routes. This can increase PLR. In SecAODV, we take into account energy and link quality in the route discovery process to create more stable paths and reduce PLR.

### 6.5. Packet Delivery Rate

This parameter is introduced as the ratio of the data packets received by the receiver to the total number of packets. This parameter is obtained according to Equation (12).


(12) $$\begin{array}{r} PDR=\frac{\overset{n}{\underset{i=1}{\sum }}P_{r}}{\overset{n}{\underset{i=1}{\sum }}P_{s}}\times 100 \end{array}$$

Where, *P*_*r*_ is packets received by the destination node and *P*_*s*_ is packets sent by the source node.

**Figure 11:**
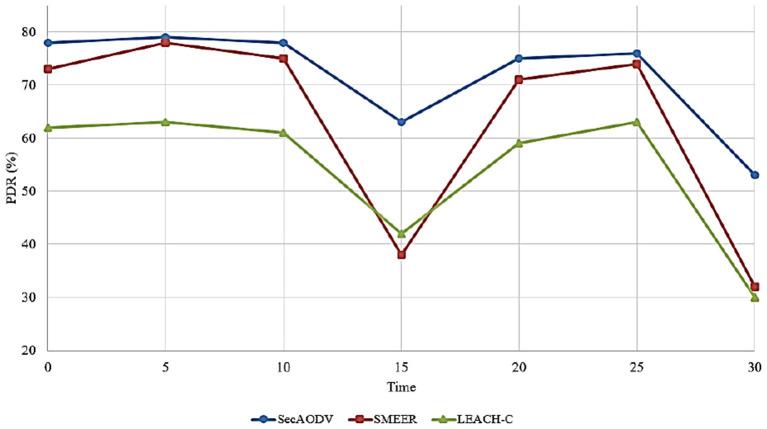
Comparison of different methods in terms of PDR.

Different routing methods have been compared in terms of PDR in Figure 11. As shown in this figure, SecAODV has the best packet delivery rate compared to other routing methods because it improves PDR by 13.83 and 32.1% compared to SMEER and LEACH-C, respectively. This shows that our proposed method facilitates the data transmission process and improves throughput. We stated its reasons in Section 6.4.

## 7. Conclusion

In this article, we proposed a secure routing scheme called SecAODV for heterogeneous WBSNs. The proposed method consists of three phases: bootstrapping, routing, and communication security. In the routing phase, we managed the RREQ replay process using a parameter called node degree. This helps SecAODV to form high-energy and high-quality paths with fewer hops between source and destination nodes. Furthermore, in the security phase, we introduced a hybrid cryptography scheme and described the key production and distribution processes. Then, SecAODV was simulated using the NS2 simulator. Finally, its results were compared with SMEER and LEACH-C in terms of end-to-end delay, throughput, energy consumption, packet delivery rate, and packet loss rate. The simulation results show that SecAODV outperforms SMEER and LEACH-C. Our scheme reduces energy consumption and delay in the data transfer process. Also, it improves throughput and provides high PDR. In future research, we attempt to strengthen security in SecAODV using powerful key encryption techniques. Also, we add an authentication mechanism to SecAODV in healthcare to prevent false packet injection or control message modification by attackers in the route discovery process. In addition, secure routing methods can be designed using some artificial intelligence techniques such as artificial neural networks (ANN), machine learning (ML) techniques, and evolutionary algorithms (EA) to achieve good results.

## Data Availability Statement

The original contributions presented in the study are included in the article/supplementary material, further inquiries can be directed to the corresponding author/s.

## Author Contributions

All authors listed have made a substantial, direct, and intellectual contribution to the work and approved it for publication.

## Conflict of Interest

The authors declare that the research was conducted in the absence of any commercial or financial relationships that could be construed as a potential conflict of interest.

## Publisher's Note

All claims expressed in this article are solely those of the authors and do not necessarily represent those of their affiliated organizations, or those of the publisher, the editors and the reviewers. Any product that may be evaluated in this article, or claim that may be made by its manufacturer, is not guaranteed or endorsed by the publisher.
